# miR-380-3p promotes β-casein expression by targeting **α_S1_**-casein in goat mammary epithelial cells

**DOI:** 10.5713/ab.23.0007

**Published:** 2023-05-04

**Authors:** Ning Song, Jun Luo, Lian Huang, Xiaoying Chen, Huimin Niu, Lu Zhu

**Affiliations:** 1 Shaanxi Key Laboratory of Molecular Biology for Agriculture, College of Animal Science and Technology, Northwest A&F University, Yangling 712100, China; 2 College of Animal Science and Technology, Anhui Agricultural University, Hefei 230036, China

**Keywords:** α_S1_-Casein, Goat Mammary Epithelial Cells, Milk Allergy, miR-380-3p

## Abstract

**Objective:**

α_S1_-Casein is more closely associated with milk allergic reaction than other milk protein components. microRNA (miRNA) is a class of small non-coding RNAs that modulate multiple biological progresses by the target gene. However, the post-transcriptional regulation of α_S1_-casein expression by miRNA in ruminants remains unclear. This study aims to explore the regulatory roles of miR-380-3p on α_S1_-casein synthesis in goat mammary epithelial cells (GMEC).

**Methods:**

α_S1_-Casein gene and miR-380-3p expression was measured in dairy goat mammary gland by quantitative real-time polymerase chain reaction (qRT-PCR). miR-380-3p overexpression and knockdown were performed by miR-380-3p mimic or inhibitor in GMEC. The effect of miR-380-3p on αS1-casein synthesis was detected by qRT-PCR, western blot, luciferase and chromatin immunoprecipitation assays in GMEC.

**Results:**

Compared with middle-lactation period, α_S1_-casein gene expression is increased, while miR-380-3p expression is decreased during peak-lactation of dairy goats. miR-380-3p reduces α_S1_-casein abundance by targeting the 3′-untranslated region (3′UTR) of α_S1_-casein mRNA in GMEC. miR-380-3p enhances β-casein expression and signal transducer and activator of transcription 5a (STAT5a) activity. Moreover, miR-380-3p promotes β-casein abundance through target gene α_S1_-casein, and activates β-casein transcription by enhancing the binding of STAT5 to β-casein gene promoter region.

**Conclusion:**

miR-380-3p decreases α_S1_-casein expression and increases β-casein expression by targeting α_S1_-casein in GMEC, which supplies a novel strategy for reducing milk allergic potential and building up milk quality in ruminants.

## INTRODUCTION

Milk protein is a dietary protein source that supplies essential amino acids and bioactive peptides for humans, mainly composed of caseins and whey proteins. Caseins account for about 80% of the total milk protein, and are divided into α_S1_-, α_S2_-, β- and κ-casein. Despite its benefits, milk protein also carries a risk of food allergies for many people, especially for infants and children [1]. The global prevalence of milk protein allergy was reported to be as high as 2% to 7.5% [2]. Among the numerous components of milk protein, α_S1_-casein is most prone to cause allergic reactions, and approximately 65% of patients with milk allergies are caused by α_S1_-casein [3]. Goat milk contains a variable content of α_S1_-casein (4% to 26% of milk protein) compared to cow milk (36% to 40%) [4]. Therefore, the method of regulating α_S1_-casein synthesis to inhibit the allergic potential of milk protein is necessary to be elucidated.

MicroRNA (miRNA) is a class of small non-coding RNA of about 22 nucleotides in length, which regulate mRNA expression by targeting the 3′-untranslated region (3′UTR) of gene transcripts. The pairing between miRNA seed site sequence (2nd to 8th nucleotides at the 5′ terminal) and target genes is considered a key feature for mammalian miRNA to recognize its targets. The abundance of miR-380-3p is involved in the prevention and treatment of a variety of human diseases. miR-380-3p participates in intracorporal neurodegeneration induced by neurotoxicants via specificity protein 3 signaling pathway [5]. High expression of miR-380-3p inhibits cell proliferation and invasion by targeting oncogene Kruppel-like factor 4 in triple negative breast cancer cells [6]. miR-380-3p increases the anti-proliferative and pro-apoptotic effects of doxorubicin in multiple models of high-risk neuroblastoma [7].

To address the milk allergy caused by α_S1_-casein in ruminants, it is urgent to investigate the regulatory mechanism of miRNAs on α_S1_-casein synthesis. Although multiple studies have revealed that miR-380-3p plays the pivotal role in a variety of biological processes, whether it could modulate ruminant milk protein expression is unknown. α_S1_-Casein gene was targeted by miR-380-3p in dairy goats or cows via TargetScan database ( http://www.targetscan.org/) prediction. Hence, the aim of our study was to explore the molecular mechanism by which miR-380-3p controls milk protein synthesis through the target gene in goat mammary epithelial cells (GMEC). In this study, we revealed that miR-380-3p reduced α_S1_-casein synthesis and enhanced β-casein abundance in GMEC, which supplies a novel approach to weaken milk allergy potential and promote milk nutritional quality in ruminants.

## MATERIALS AND METHODS

### Animal care

The animal study protocol was approved by the Institutional Animal Care and Use Committee, Northwest A&F University, YangLing, China (protocol code 15–516).

### Mammary tissue collection and cell culture

Mammary gland samples were obtained from 3-year-old healthy Xinong Saanen dairy goats (n = 5) during early, peak, middle and end lactation periods (15, 60, 120, and 270 days postpartum, respectively). GMEC were isolated from three dairy goats at peak lactation period. Cells were cultured in the incubator at 37°C and 5% CO_2_. The culture medium contained 90% DMEM/F12 (SH30023; Hyclone, Logan, UT, USA) medium and 10% fetal bovine serum (10099141; Gibco, Gaithersburg, MD, USA), with 5 μg/mL bovine insulin (16634; Sigma, St. Louis, MO, USA), 1 μg/mL hydrocortisone (H0888; Sigma, USA), 10 ng/mL epidermal growth factor (PHG0311; Invitrogen, Carlsbad, CA, USA), 100 U/mL penicillin/streptomycin (080092569; Harbin Pharmaceutical Group, Harbin, China) added.

### Vector construction

Primers for sequence cloning of goat α_S1_-casein gene (GenBank no. XM_018049127.1) and β-casein promoter region (GenBank no. NC_030813.1) are shown in Supplementary Table S1. The template of coding sequence and 3′UTR region cloning of α_S1_-casein was cDNA obtained from goat mammary gland. The template of β-casein promoter region cloning was goat blood genomic DNA. pcDNA3.1-α_S1_-casein vector was constructed using goat α_S1_-casein coding sequence and pcDNA3.1 plasmid. psi-WT-α_S1_-Casein vector was produced using α_S1_-casein 3′UTR sequence and psiCHECK2 plasmid. α_S1_-Casein 3′UTR site-directed mutant vector psi-MUT-α_S1_-Casein was produced using overlapping polymerase chain reaction (PCR) method. pGL3-WT-β-Casein vector was constructed using β-casein promoter sequence and pGL3-Basic plasmid. Similarly, β-casein promoter site-directed mutant vector was performed and named pGL3-MUT-β-Casein. All constructed vectors were sequenced to confirm the sequences.

### Cell treatment

α_S1_-Casein 3′UTR was targeted by miR-380-3p using TargetScan database prediction., The experimental treatments were carried out when cell confluence reached 80%. For miR-380-3p overexpression, cells were transfected with miR-380-3p mimic or the mimic negative control (NC) (50 nM; RiboBio, Guangzhou, China) using Lipofectamine RNAiMAX (13778150; Invitrogen, USA). Similarly, for miR-380-3p knockdown, GMEC were treated with miR-380-3p inhibitor or the negative control inhibitor NC (100 nM; RiboBio, China). We silenced αS1-casein gene by specific small interference RNA (si-α_S1_-Casein; GenePharma, Shanghai, China). The sequences of si-α_S1_-Casein and the negative control si-NC are shown in Supplementary Table S2. GMEC were co-treated with si-α_S1_-Casein (100 nM) and miR-380-3p inhibitor (100 nM) using Lipofectamine RNAiMAX. α_S1_-Casein overexpression was carried out by pcDNA3.1-α_S1_-casein plasmid. Cells were co-treated with pcDNA3.1-α_S1_-casein and miR-380-3p mimic (50 nM) using X-treme GENE HP Regent (06366236001; Roche, Mannheim, Germany). GMEC were collected after 48 h treatment to extract RNA and protein.

### Quantitative real-time polymerase chain reaction assay

Total RNA of cells was extracted by RNAiso Plus (9109; Takara, Shiga, Japan). The quality of total RNA was detected by NanoDrop2000 spectrophotometer (Thermo Scientific, Waltham, MA, USA). The optical density 260/280 of total RNA was 1.9 to 2.1, and the concentration was approximately 500 ng/μL. For gene quantitative analysis, cDNA was synthesized by purified total RNA with PrimeScript RT kit (RR047A; Takara, Japan). Gene quantitative real-time PCR (qRT-PCR) was carried out with TB Green II kit (RR820A; Takara, Japan). The control genes were ubiquitously expressed transcript (*UXT*) and ribosomal protein S9 (*RPS9*). Primers for gene quantitative analysis are listed in Table 1. For miR-380-3p quantitative analysis, cDNA of mature miR-380-3p was produced by miRcute cDNA kit (KR211; Tiangen, Beijing, China). qRT-PCR of miR-380-3p were performed by miRcute q-PCR kit (FP411; Tiangen, China). The control for miR-380-3p was 5S rRNA. The quantitative primer sequence of miR-380-3p was 5′-CGCTATGTAATGTGGTCC ACGTCT-3′. The primer sequence of 5S rRNA was 5′-TCTC AGAAGCTAAACAGGGTCG-3′. All qRT-PCR experiments were carried out by Bio-Rad CFX96 analyzer (Hercules, CA, USA). Quantitative data were analyzed by 2^−ΔΔCt^ method.

### Western blot assay

Total protein of cells was collected by radioimmunoprecipitation assay lysis regent (R0010; Solarbio, Beijing, China) with protease and phosphatase inhibitor (04693132001 and 04906845001; Roche, Germany). The concentration of total protein was detected by bicinchoninic acid assay kit (23227; Thermo Scientific, USA). The protein was separated by 10% sodium dodecyl sulfate-polyacrylamide gel electrophoresis and blotted onto the polyvinylidene fluoride membrane (Roche, Germany) with Bio-Rad Trans-Blot semidry transfer. Nonspecific sites were blocked using 5% skim milk (232100; BD Biosciences, Franklin Lakes, NJ, USA) at least 2 h, then incubated at least 12 h at 4°C with primary antibody: rabbit α_S1_-casein (SAB1401093; Sigma-Aldrich, USA; 1:1,000), rabbit β-casein (orb2053; Biorbyt, Cambridge, UK; 1:500), mouse signal transducer and activator of transcription 5a (STAT5a) (610191; BD Biosciences, USA; 1:1,000), mouse p-STAT5a (611964; BD Biosciences, USA; 1:500), or mouse β-actin (CW0096; CW Biotech, Beijing, China; 1:2,000). Secondary antibody was horseradish peroxidase-conjugated goat anti-mouse-immunoglobulin G (IgG) (CW0102; CW Biotech, China; 1:5,000) or goat anti-rabbit-IgG (CW0103; CW Biotech, China; 1:5,000). Blots were detected using chemiluminescent kit (1705061; Bio-Rad, USA). The blot intensity was analyzed by ImageJ ( http://imagej.nih.gov/ij/). Expression of caseins was normalized by comparison with β-actin, and expression of p-STAT5a was normalized to STAT5a.

### Luciferase assay

For activity assay of 3′UTR, GMEC were co-treated with α_S1_-casein 3′UTR vectors and miR-380-3p mimic (50 nM) by X-treme GENE HP Regent. For activity assay of promoter, cells were co-treated with β-casein promoter vectors and miR-380-3p mimic (50 nM). β-Casein promoter vector was treated along with pRL-TK renilla vector as an internal control. Also, GMEC were treated with STAT5 inhibitor STAT5-IN-1 (50 μM, CAS 285986-31-4; MedChemExpress, NJ, USA) followed by miR-380-3p mimic (50 nM) and psi-WT-β-Casein co-treatment. After 48 h treatment, cells were collected and lysed for luciferase assay. Data were measured using a Dual-Luciferase System (E1910; Promega, Madison, WI, USA) by Fluoroskan Ascent (Thermo Scientific, USA). The luciferase activity of 3′UTR was analyzed as the ratio of renilla compared with firefly luciferase activity. The promoter activity was analyzed as the ratio of firefly compared with renilla activity.

### Chromatin immunoprecipitation assay

The chromatin immunoprecipitation (ChIP) assay was carried out by Beyotime kit (P2048; Shanghai, China). Cells were cultured in 100-mm dish and treated with miR-380-3p mimic (50 nM) or STAT5-IN-1 (50 μM) for 48 h. Then, cells were crosslinked by 1% formaldehyde (final concentration) for 10 min at 37°C and harvested in cold PBS with protease and phosphatase inhibitor. Chromosomal DNA was sheared to lengths of 100 to 500 bp by Bioruptor Pico (Diagenode, Seraing, Belgium). The chromatin DNA-protein compound was incubated with protein A/G beads and rabbit p-STAT5 (9351S; Cell Signaling Technology, Danvers, MA, USA; 1:50) or control rabbit IgG (A7016; Beyotime, China; 1:50) for 12 h at 4°C. The primers containing STAT5 binding site on β-casein promoter region were shown in Table 1.

### Statistical analysis

All experiments were repeated for at least for 3 biological replicates. Data were shown as mean±standard error of the mean and analyzed by SPSS 20.0 software (IBM, Chicago, IL, USA). T-test was carried out with two group data, and one-way analysis of variance with Tukey test was carried out for multiple comparison. The difference was significant when p<0.05 (* p<0.05, ** p<0.01).

## RESULTS

### Expression of **α_S1_**-casein and miR-380-3p in different lactation periods

**Figure 1:**
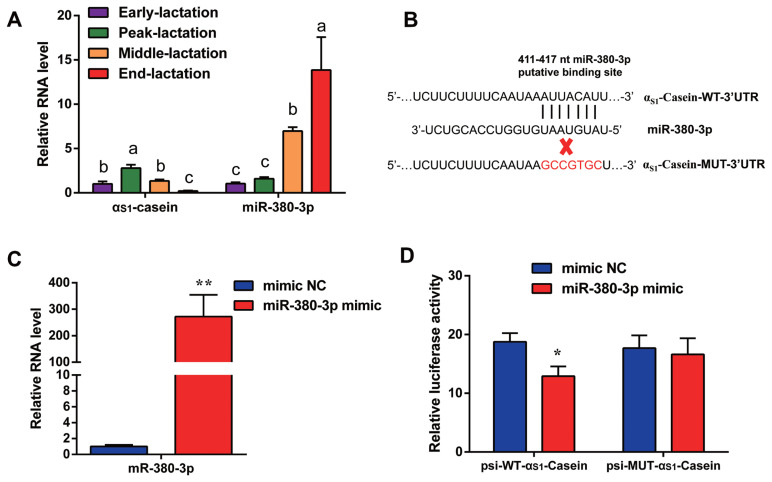
miR-380-3p targets 3′UTR of α_S1_-casein gene. (A) α_S1_-Casein gene and miR-380-3p abundance in goat mammary gland of different lactation stages. Mammary gland samples were obtained from Xinong Saanen dairy goats (n = 5) during early, peak, middle and end lactation stages. Values were normalized to early lactation stage. (B) Predicted binding site of miR-380-3p in α_S1_-casein 3′UTR and construction of psiCHECK-2 dual-luciferase reporter vectors. psi-WT-α_S1_-casein represents the wildtype sequence vector; psi-MUT-α_S1_-casein represents the mutation (red bases) vector. (C) miR-380-3p expression was measured by qRT-PCR. GMEC were treated with mimic NC or miR-380-3p mimic (50 nM) for 48 h. (D) 3′UTR activity of α_S1_-casein was measured by luciferase assay. Cells were co-treated with dual-luciferase vector and miR-380-3p mimic (50 nM) for 48 h. Data are presented as mean±standard error of the mean. UTR, untranslated region; qRT-PCR, quantitative real-time polymerase chain reaction; GMEC, goat mammary epithelial cells; NC, negative control. ^a–c^ Different superscripts represent significant differences among α_S1_-casein or miR-380-3p levels (p<0.05). * p<0.05 and ** p<0.01 compared with control.

Quantitative result in goat mammary tissue showed that α_S1_-casein gene expression was highest in peak of lactation and lowest in end of lactation stage (Figure 1A). Based on TargetScan database prediction, miR-380-3p targets 3′UTR of goat α_S1_-casein gene (Figure 1B). Expression of miR-380-3p was higher in end-lactation than other lactation stages. Specifically, α_S1_-casein gene expression was 2.13-fold higher in peak-lactation than middle-lactation stage (p<0.05), while miR-380-3p expression in middle-lactation was 4.39-fold higher than peak-lactation stage (p<0.05; Figure 1A).

### miR-380-3p targets 3′UTR of **α_S1_**-casein gene

To explore whether miR-380-3p did directly target 3′UTR of α_S1_-casein, cells were treated with miR-380-3p mimic. Compared with mimic NC, miR-380-3p expression was up-regulated 272-fold in miR-380-3p mimic group (p<0.01; Figure 1C). The TargetScan database predicted that miR-380-3p binds to the bases at positions 411 to 417 of α_S1_-casein 3′UTR (Figure 1B). Therefore, we detected dual-luciferase activity of wildtype and mutant plasmids after overexpressing miR-380-3p. Luciferase assay revealed that miR-380-3p could directly bind to specific site of α_S1_-casein 3′UTR in dairy goats (Figure 1D).

### miR-380-3p decreases **α_S1_**-casein expression

**Figure 2:**
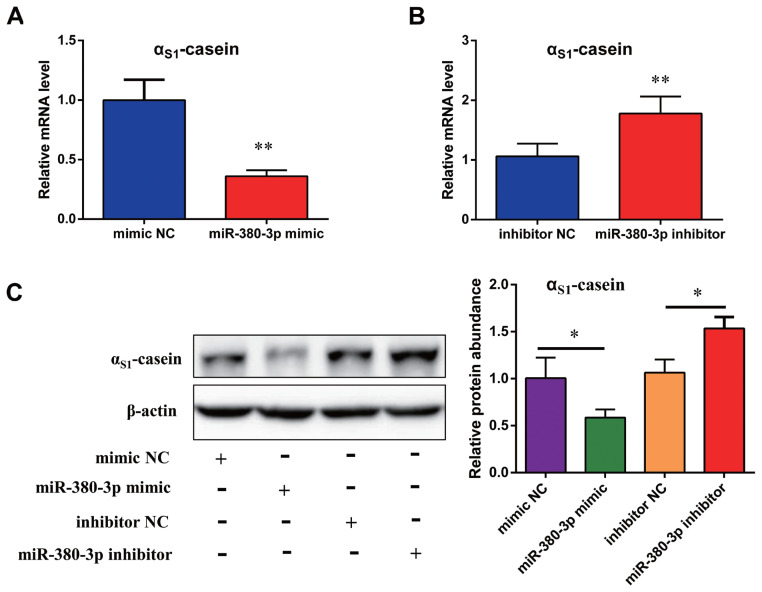
miR-380-3p inhibits α_S1_-casein expression. (A) Expression of α_S1_-casein gene in cells treated with miR-380-3p mimic (50 nM) for 48 h. (B) Expression of α_S1_-casein gene in cells treated with miR-380-3p inhibitor (100 nM) for 48 h. (C) Expression of α_S1_-casein in cells treated with miR-380-3p mimic (50 nM) or miR-380-3p inhibitor (100 nM) for 48 h. Relative α_S1_-casein expression was normalized by comparison with β-actin. Data are presented as mean±standard error of the mean. * p<0.05 and ** p<0.01 compared with control.

Next, to confirm the effect of miR-380-3p on expression of α_S1_-casein, cells were transfected with mimic or inhibitor of miR-380-3p. Compared with mimic NC, expression of α_S1_-casein gene was significant reduced by an average of 64% in miR-380-3p mimic group (p<0.01; Figure 2A). Compared with inhibitor NC, expression of α_S1_-casein gene was effectively enhanced by an average of 78% in miR-380-3p inhibitor group (p<0.01; Figure 2B). Further, compared with control, miR-380-3p mimic could decrease α_S1_-casein expression by an average of 41% (p<0.05; Figure 2C). On the contrary, α_S1_-casein expression was increased by an average of 54% after miR-380-3p inhibition (p<0.05; Figure 2C). These finds suggest that miR-380-3p decreases α_S1_-casein expression which is strongly related to milk allergy potential.

### miR-380-3p enhances STAT5a activity and **β**-casein abundance

**Figure 3:**
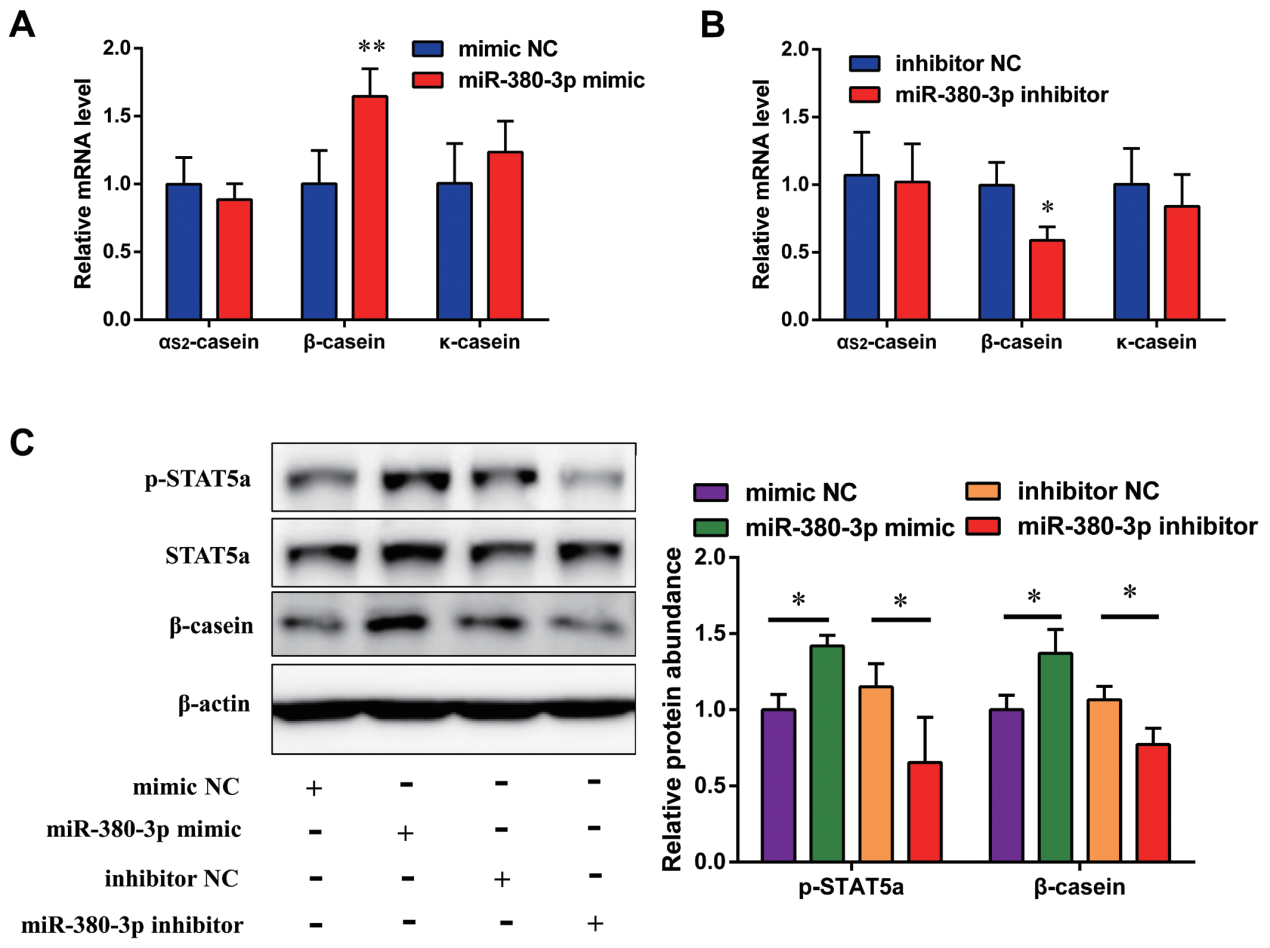
miR-380-3p increases STAT5a activity and β-casein abundance. (A) Expression of α_S2_-casein, β-casein and κ-casein gene in cells treated with miR-380-3p mimic (50 nM) for 48 h. (B) Expression of α_S2_-casein, β-casein and κ-casein gene in cells treated with miR-380-3p inhibitor (100 nM) for 48 h. (C) Expression of p-STAT5a and β-casein in cells treated with miR-380-3p mimic (50 nM) or miR-380-3p inhibitor (100 nM) for 48 h. Relative β-casein expression was normalized by comparison with β-actin. Relative p-STAT5a expression was normalized to STAT5a. STAT5a, signal transducer and activator of transcription 5a. Data are presented as mean±standard error of the mean. * p<0.05 and ** p<0.01 compared with control.

To investigate whether miR-380-3p affects expression of other caseins, we measured the mRNA levels of α_S2_-casein, β-casein, and κ-casein gene. Results showed that overexpressing miR-380-3p increased β-casein gene expression (p<0.01; Figure 3A), but could not regulate α_S2_-casein and κ-casein mRNA levels. Similarly, knockdown of miR-380-3p decreased expression of β-casein gene as well (p<0.05; Figure 3B). On the other hand, α_S2_-casein and κ-casein mRNA levels had no significant difference between miR-380-3p inhibitor and inhibitor NC groups (Figure 3B). Then, we measured the expression of β-casein and STAT5a, a key transcription factor for milk protein synthesis. Results showed that miR-380-3p mimic markedly upregulated abundance of STAT5a phosphorylation (p-STAT5a) and β-casein (p<0.05; Figure 3C). Uniformly, miR-380-3p knockdown significantly reduced p-STAT5a and β-casein abundance (p<0.05; Figure 3C). Collectively, these results suggest that miR-380-3p decreases α_S1_-casein expression and upregulates β-casein expression, which is beneficial to hypoallergenic goat milk.

### miR-380-3p promotes **β**-casein expression by targeting **α_S1_**-casein

**Figure 4:**
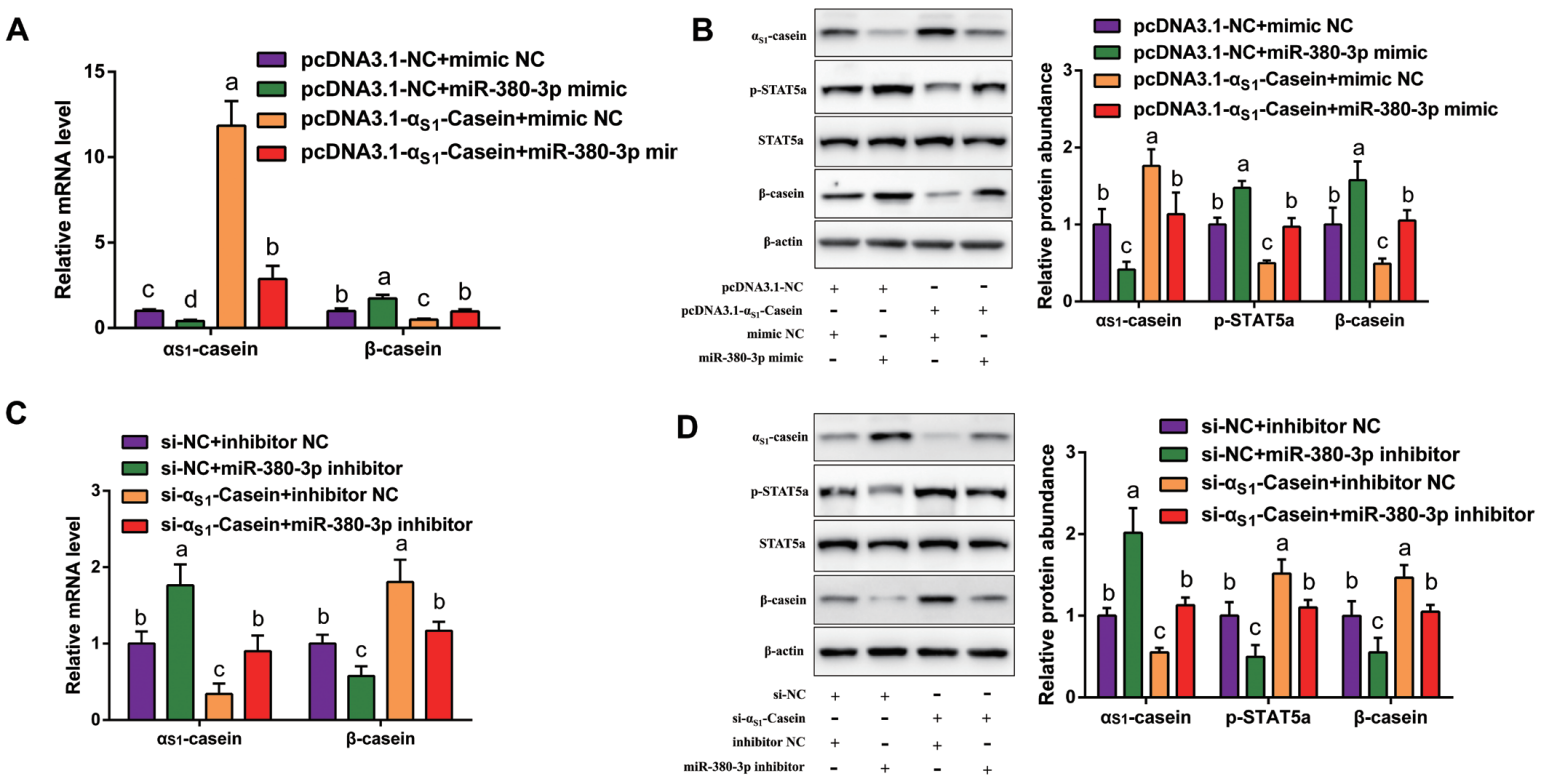
miR-380-3p improves β-casein expression by targeting α_S1_-casein gene. (A, B) Expression of α_S1_-casein, p-STAT5a and β-casein in cells co-treated with pcDNA3.1-α_S1_-casein and miR-380-3p mimic (50 nM) for 48 h. (C, D) Expression of α_S1_-casein, p-STAT5a and β-casein in cells co-treated with si-α_S1_-Casein (100 nM) and miR-380-3p inhibitor (100 nM) for 48 h. Relative expression of α_S1_-casein and β-casein was normalized by comparison with β-actin. Relative p-STAT5a expression was normalized to STAT5a. STAT5a, signal transducer and activator of transcription 5a. Data are presented as mean±standard error of the mean. ^a–c^ Different superscripts represent significant differences among groups (p<0.05).

Our previous study discovered that α_S1_-casein gene decreased β-casein expression by inhibiting STAT5a activity in GMEC [8]. To confirm whether miR-380-3p increased β-casein expression through α_S1_-casein/STAT5a regulation axis, cells were co-transfected with miR-380-3p mimic and pcDNA3.1-α_S1_-casein. Compared with mimic NC + pcDNA3.1-NC group, α_S1_-casein expression was markedly upregulated, while p-STAT5a and β-casein expression was decreased in mimic NC + pcDNA3.1-α_S1_-casein group (p<0.05; Figure 4A, B). Compared with mimic NC + pcDNA3.1-α_S1_-casein group, α_S1_-casein expression was significantly decreased, while p-STAT5a and β-casein expression was increased in miR-380-3p mimic + pcDNA3.1-α_S1_-casein group (p<0.05; Figure 4A, B).

Similarly, silencing α_S1_-casein reduced α_S1_-casein abundance, with the increase of p-STAT5a and β-casein expression (p<0.05; Figure 4C, D). Furthermore, when we knocked down both α_S1_-casein and miR-380-3p, the increase in p-STAT5a and β-casein expression caused by α_S1_-casein silencing was abolished as a result of miR-380-3p inhibition (Figure 4C, D). Taken together, we deduce that miR-380-3p indirectly increases β-casein expression and STAT5 activity which leans upon α_S1_-casein reduction.

### miR-380-3p improves **β**-casein transcription via upregulating STAT5 activity

**Figure 5:**
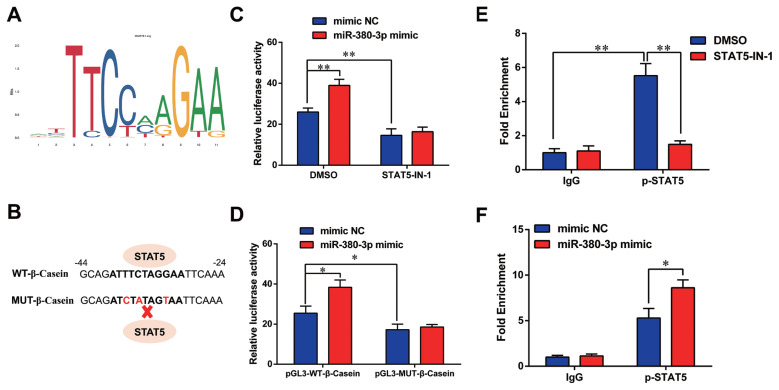
miR-380-3p activates β-casein transcription via regulating STAT5. (A) Conserved binding motif of transcription factor STAT5. (B) Binding site of STAT5 in β-casein promoter region and construction of pGL3 luciferase reporter vectors. pGL3-WT-β-casein represents the wildtype β-casein promoter vector; pGL3-MUT-β-Casein represents the mutation (red bases) vector on STAT5 binding sequence. (C) Promoter activity of β-casein was measured by luciferase assay. Cells were treated with STAT5-IN-1 (50 μM) followed by pGL3-WT-β-Casein and miR-380-3p mimic (50 nM) co-treatment for 48 h. (D) Relative luciferase activity in cells co-treated with pGL3-β-Casein and miR-380-3p mimic (50 nM) for 48 h. (E) ChIP assay of the STAT5 binding site to β-casein gene promoter by qRT-PCR. Cells were treated with STAT5-IN-1 (50 μM) for 48 h. (F) ChIP assay in cells treated with miR-380-3p mimic (50 nM) for 48 h. STAT5a, signal transducer and activator of transcription 5a; qRT-PCR, quantitative real-time polymerase chain reaction. Data are presented as mean±standard error of the mean. * p<0.05 and ** p<0.01 compared with control.

Previous studies have shown STAT5 enhances β-casein transcription by binding to β-casein promoter region in dairy goats [9]. Therefore, we determined whether miR-380-3p modulated β-casein transcription through STAT5. Figure 5A shows the conserved binding motif of transcription factor STAT5. We constructed the wild-type and mutant luciferase plasmids of β-casein promoter according to STAT5 binding site (Figure 5B). miR-380-3p obviously increased β-casein promoter activity, but STAT5 inhibitor reduced β-casein promoter activity regardless of miR-380-3p overexpression (p<0.01; Figure 5C). Consistently, compared with pGL3-WT-β-Casein promoter group, pGL3-MUT-β-Casein promoter activity was markedly weakened regardless of miR-380-3p overexpression (p<0.05; Figure 5D).

**Figure 6:**
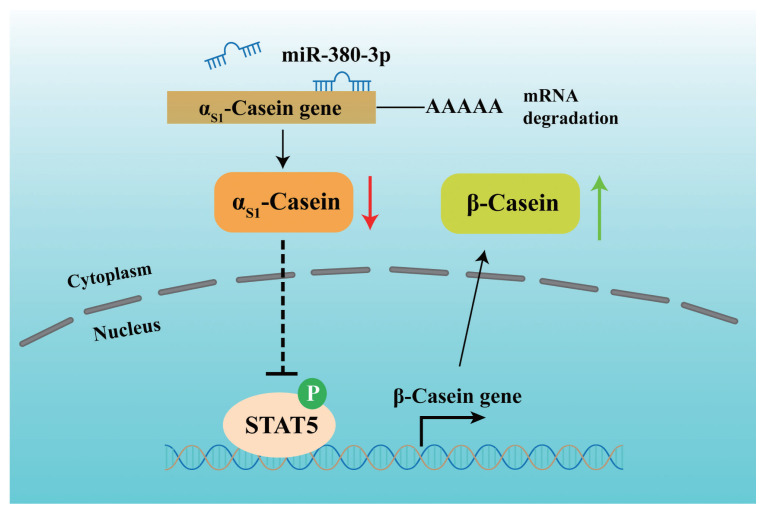
The proposed model regarding the role and regulation of miR-380-3p in goat mammary epithelial cells. miR-380-3p inhibits αS1-casein expression by targeting 3′UTR of αS1-casein gene. Then, miR-380-3p increases β-casein expression through αS1-casein/STAT5 axis. Moreover, miR-380-3p promotes β-casein transcription by the binding of STAT5 to β-casein gene promoter region. STAT5a, signal transducer and activator of transcription 5a.

Further, we attempted to identify the binding of STAT5 to β-casein promoter by ChIP analysis. Compared with rabbit IgG control group, STAT5 forcefully bound to the specific site of β-casein promoter region in p-STAT5 group (p<0.01), but STAT5 inhibitor highly attenuated the combination of STAT5 and β-casein promoter region (p<0.01; Figure 5E). Subsequently, we discovered that the binding efficiency of STAT5 and β-casein promoter was significantly enhanced by miR-380-3p mimic treatment (p<0.05; Figure 5F). These data declared that STAT5 activated β-casein transcription and that the modulation of STAT5 on β-casein promoter was increased by miR-380-3p overexpression. Overall, our study revealed that miR-380-3p inhibits α_S1_-casein expression by targeting α_S1_-casein, and indirectly promotes β-casein transcription via α_S1_-casein/STAT5 axis in GMEC (Figure 6).

## DISCUSSION

Understanding the regulatory mechanism of miRNA in physiological progresses could provide the theoretical basis for its application in animals. Previous studies have shown miRNAs modulate the synthesis process of milk proteins. miR-424-5p targeted 3′UTR of β-casein gene by prolactin receptor signaling pathway in murine mammary glands [10]. miR-2904 positively regulated milk protein synthesis via Janus kinase 2 (JAK2)/STAT5 signaling pathway by the functional network analysis of Chinese Holstein cows [11]. In bovine mammary epithelial cells (BMEC), miR-139 inhibited β-casein expression through targeting growth hormone receptor and insulin-like growth factor receptor [12]. miR-27a negatively regulated milk protein and lactose contents through targeting mitogen-activated protein kinase 14 that had a positive effect on milk protein expression in sheep [13]. In GMEC, miR-8516 promoted the secretion of α_S1_-casein and β-casein by activating the activity of JAK2/STAT5 [14]. Although these studies indicate the miRNA regulation of milk protein synthesis, research on miRNA-targeted α_S1_-casein expression are still insufficient. According to the TargetScan software prediction, miR-380-3p targets 3′UTR region of goat α_S1_-casein gene. A dual-luciferase experiment was carried out to identify the targeted relationship of miR-380-3p to α_S1_-casein gene. More importantly, miR-380-3p decreased the expression of α_S1_-casein gene and α_S1_-casein in dairy goats, which contributed to low allergy potential in goat milk.

miR-380-3p is involved in multiple biological progresses, such as cell proliferation, apoptosis, inflammation and melanogenesis. miR-380-3p targeted sex determining region Y-box 6 to regulate melanogenesis by affecting beta-catenin and microphthalmia-associated transcription factor expression in melanocytes [15]. High expression of miR-380-3p increased cell inflammation by promoting tumor necrosis factor-alpha, interleukin-1 beta, and interleukin-18 levels in spinal cord jury model rats [16]. Overexpression of miR-380-3p activated inflammation, oxidative stress, and apoptosis by targeting suppressor of cytokine signaling 6 in retinal epithelial cells with diabetic retinopathy [17]. In mouse neuroblastoma cells, miR-380-3p inhibited cell proliferation and increased cell apoptosis rate through blocking the translation of transcription factor specificity protein-3 [18]. On the contrary, miR-380-3p, acted as an oncogenic miRNA, promoted cell proliferation, epithelial-mesenchymal transition, and tumorigenesis in pancreatic cancer cells [19]. Despite the potent features of miR-380-3p, its regulatory role in milk protein expression is still unknown. In our research on dairy goats, we uncovered that miR-380-3p decreased α_S1_-casein expression and enhanced β-casein abundance by targeting 3′UTR of α_S1_-casein gene. We expected that there was a correlation between α_S1_-casein and β-casein abundance regulated by miR-380-3p, which should be investigated in more detail in GMEC.

Multiple studies have shown the association between the expression of different milk protein components. In mouse mammary epithelial cells, lipoteichoic acid treatment increased the intracellular and secreted α_S1_-casein accompanied by a decrease in β-casein content [20]. Compared with wild-type dairy cows, the contents of α_S1_-casein, β-casein, κ-casein and α-lactalbumin were all increased in β-lactoglobulin silenced cows [21]. On the contrast, the contents of α-casein and α-lactalbumin in milk from β-casein and κ-casein transgenic cows were lower than non-transgenic cows [22]. There was a negative correlation between α_S1_-casein and β-casein gene expression in BMEC treated with camellia seed oil [23]. In mammary gland of lactation goats, α_S1_-casein gene expression was negatively correlated with β-lactoglobulin mRNA level [24]. More importantly, our previous study found that α_S1_-casein gene negatively regulated STAT5a phosphorylation and β-casein expression in GMEC [8]. In this research on GMEC, we carried out rescue assays to identify whether miR-380-3p up-regulated p-STAT5a and β-casein abundance by targeting α_S1_-casein gene. miR-380-3p mimic rescued the inhibition of α_S1_-casein gene on p-STAT5a and β-casein expression, while miR-380-3p inhibitor moderated the impact of α_S1_-casein gene knockdown on p-STAT5a and β-casein abundance. These rescue assays reveal that miR-380-3p indirectly increases STAT5 activity and β-casein expression by inhibiting α_S1_-casein expression. The present results are supported by our previous study that α_S1_-casein downregulated STAT5 activity and β-casein expression.

Previous studies have shown STAT5 is a primary transcriptional factor for milk production in mammals. STAT5 binds to milk protein gene promoters by identifying the specific binding motif. In STAT5 knockout mouse model, there was a significant reduction in the content of α-lactalbumin and β-lactoglobulin in milk [25]. Curcumin and basolateral lactose exposure suppressed intracellular and secreted casein production by STAT5 inactivation in mouse mammary epithelial cells [26]. In BMEC, beta-sitosterol promoted β-casein synthesis through the activation of STAT5 [27]; biochanin A declined β-casein synthesis and secretion by inhibiting STAT5 activity [28]. Milk protein expression was markedly decreased when STAT5a activity was blocked by suppressor of cytokine signaling 3 in dairy cows [29]. Further, phosphorylated STAT5a bound to β-casein promoter region and increased β-casein synthesis in immortalized GMEC [30]. Consistent with previous studies, luciferase experiments have shown that phosphorylated STAT5 promoted β-casein gene transcription as well in the present study. More importantly, ChIP experiments were carried out to demonstrate the direct binding of STAT5 to β-casein gene promoter, which could be activated by miR-380-3p mimic. Therefore, miR-380-3p indirectly promotes β-casein transcription through α_S1_-casein/STAT5a regulation axis. Additionally, α_S1_-casein gene could be targeted by miR-380-3p both in dairy goats and cows via TargetScan database prediction. In this study, we verified that miR-380-3p targets α_S1_-casein gene and promotes STAT5 activity and β-casein expression by inhibiting α_S1_-casein expression in dairy goats. However, whether miR-380-3p could target α_S1_-casein gene and play similar regulatory roles needs to be further studied in dairy cows.

## CONCLUSION

miR-380-3p targets 3′UTR of goat α_S1_-casein gene and inhibits α_S1_-casein expression which is associated with higher rates of milk allergy for humans. miR-380-3p promotes β-casein expression through targeting α_S1_-casein gene. Moreover, miR-380-3p increases β-casein transcription by the binding of STAT5 to β-casein gene promoter region. In summary, our study is helpful to conclude the regulatory role of miR-380-3p on milk protein synthesis and supplies a novel basis for declining the potential of milk allergy through regulating miR-380-3p in ruminants.

## Figures and Tables

**Table 1:** Primers used for quantitative real-time polymerase chain reaction of genes and promoter region

NCBI accession ID	Gene	Primer sequence (5′-3′)	Length (bp)
XM_018049127.1	α_S1_-casein	F, TCCACTAGGCACACAATACACTGA	61
		R, GCCAATGGGATTAGGGATGTC	
NM_001285585.1	α_S2_-casein	F, CTGGTTATGGTTGGACTGGAAAA	76
		R, AACATGCTGGTTGTATGAAGTAAAGTG	
XM_005681721.2	β-casein	F, CCCAGGCACAGTCTCTAGTCT	196
		R, GGCTCAACTGGATATTTAGGGA	
NM_001285587.1	κ-casein	F, AGGTGCAATGATGAAGAGTTTTTTC	66
		R, CCCAAAAATGGCAGGGTTAA	
XM_005700842.2	*UXT*	F, CAGCTGGCCAAATACCTTCAA	125
		R, GTGTCTGGGACCACTGTGTCAA	
XM_005709411.1	*RPS9*	F, CCTCGACCAAGAGCTGAAG	64
		R, CCTCCAGACCTCACGTTTGTTC	
NC_030813.1	β-casein promoter	F, AATAGGAAGAATTCATTTCCTA	102
		R, TATAGTATTTAATGGCATGCTA	

*UXT*, ubiquitously expressed transcript; *RPS9*, ribosomal protein S9.

## SUPPLEMENTARY MATERIAL

**Supplementary Table S1**. Primers used for amplification of αS1-casein and β-casein gene

**Supplementary Table S2**. Sequences of siRNA targeting goat αS1-casein gene

**Supplementary Figure S1**. The image of the full western blot of Figure 2C. Expression of αS1-casein in cells treated with miR-380-3p mimic (50 nM) or miR-380-3p inhibitor (100 nM) for 48 h.

**Supplementary Figure S2**. The image of the full western blot of Figure 3C. Expression of p-STAT5a and β-casein in cells treated with miR-380-3p mimic (50 nM) or miR-380-3p inhibitor (100 nM) for 48 h.

**Supplementary Figure S3**. The image of the full western blot of Figure 4B. Expression of αS1-casein, p-STAT5a and β-casein in cells co-treated with pcDNA3.1-αS1-casein and miR-380-3p mimic (50 nM) for 48 h.

**Supplementary Figure S4**. The image of the full western blot of Figure 4D. Expression of αS1-casein, p-STAT5a and β-casein in cells co-treated with si-αS1-Casein (100 nM) and miR-380-3p inhibitor (100 nM) for 48 h.
