# Supplementary material for: miR-380-3p promotes β-casein expression by targeting αS1-casein in goat mammary epithelial cells
Source: Anim Biosci. 2023 May 4;36(10):1488–98. doi: 10.5713/ab.23.0007 (PMC10475382; doi:10.5713/ab.23.0007)
Supplement: Supplementary file 1 [file ab-23-0007-Supplementary-Table-1.pdf]

Supplemental Table 1. Primers used for amplification of α_{S1}-casein and β-casein gene

primer name	primer sequence (5′-3′)	length (bp)
α_{S1}-casein (CDS clone)	F, AGCTTGCTGCTTCTTCCCAGT	695
	R, ACTCTTCACCACAGTGGCATAG	
α_{S1}-casein (pcDNA3.1 vector)	F, CCGCTCGAGGCCACCATGAAACTTCTCATCCTTAC	642
	R, CCCAAGCTTTCACCACAGTGGCATAG	
α_{S1}-casein (3'UTR clone)	F, CCGCTCGAGAGAGTCAAGTGAATTCTGAGGA	429
	R, ATAAGAATGCGGCCGCTAGTGCCTTAAAATGTAATTTATTG	
α_{S1}-casein (3'UTR mutagenesis)	mut, TAGTGCCTTAAAGCACGGCTTATTGAA	429
	anti-mut, TCTTCTTTTCAATAAGCCGTGCTTTAAG	
β-casein (promoter clone)	F, CGACGCGTTGCAAATCTCAGGACTGCAT	2402
	R, CCGCTCGAGCTGTTAACTAGTCTTGTGCT	
β-casein (promoter mutagenesis)	mut, ATAGTGGATTTGAATTACTATAGATCTGCA	2402
	anti-mut, TCCTAATCATGCAGATCTATAGTAATTCAAA	
