# Supplementary material for: miR-380-3p promotes β-casein expression by targeting αS1-casein in goat mammary epithelial cells
Source: Anim Biosci. 2023 May 4;36(10):1488–98. doi: 10.5713/ab.23.0007 (PMC10475382; doi:10.5713/ab.23.0007)
Supplement: Supplementary file 2 [file ab-23-0007-Supplementary-Table-2.pdf]

Supplemental Table 2. Sequences of siRNA targeting goat α_{S1}-casein gene

**siRNA name**	**sense sequence (5'-3')**	**antisense sequence (5'-3')**
si-NC	UUCUCCGAACGUGUCACGUTT	ACGUGACACGUUCGGAGAATT
si-α_{S1}-Casein	GCUGGACGCCUAUCCAUCUTT	AGAUGGAUAGGCGUCCAGCTT
