# Supplementary material for: miR-380-3p promotes β-casein expression by targeting αS1-casein in goat mammary epithelial cells
Source: Anim Biosci. 2023 May 4;36(10):1488–98. doi: 10.5713/ab.23.0007 (PMC10475382; doi:10.5713/ab.23.0007)
Supplement: Supplementary file 3 [file ab-23-0007-Supplementary-Fig-1.pdf]

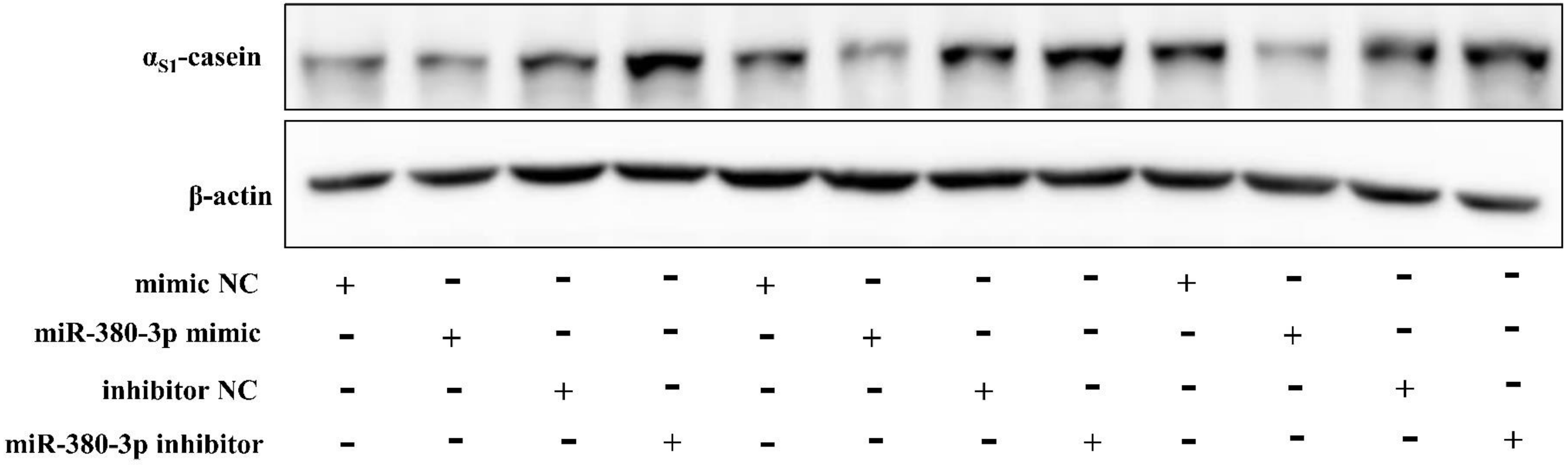

Supplemental Figure S1. The image of the full western blot of Figure 2C. Expression of α_{S1}-casein in cells treated with miR-380-3p mimic (50 nM) or miR-380-3p inhibitor (100 nM) for 48 h.
