# Supplementary material for: miR-380-3p promotes β-casein expression by targeting αS1-casein in goat mammary epithelial cells
Source: Anim Biosci. 2023 May 4;36(10):1488–98. doi: 10.5713/ab.23.0007 (PMC10475382; doi:10.5713/ab.23.0007)
Supplement: Supplementary file 5 [file ab-23-0007-Supplementary-Fig-3.pdf]

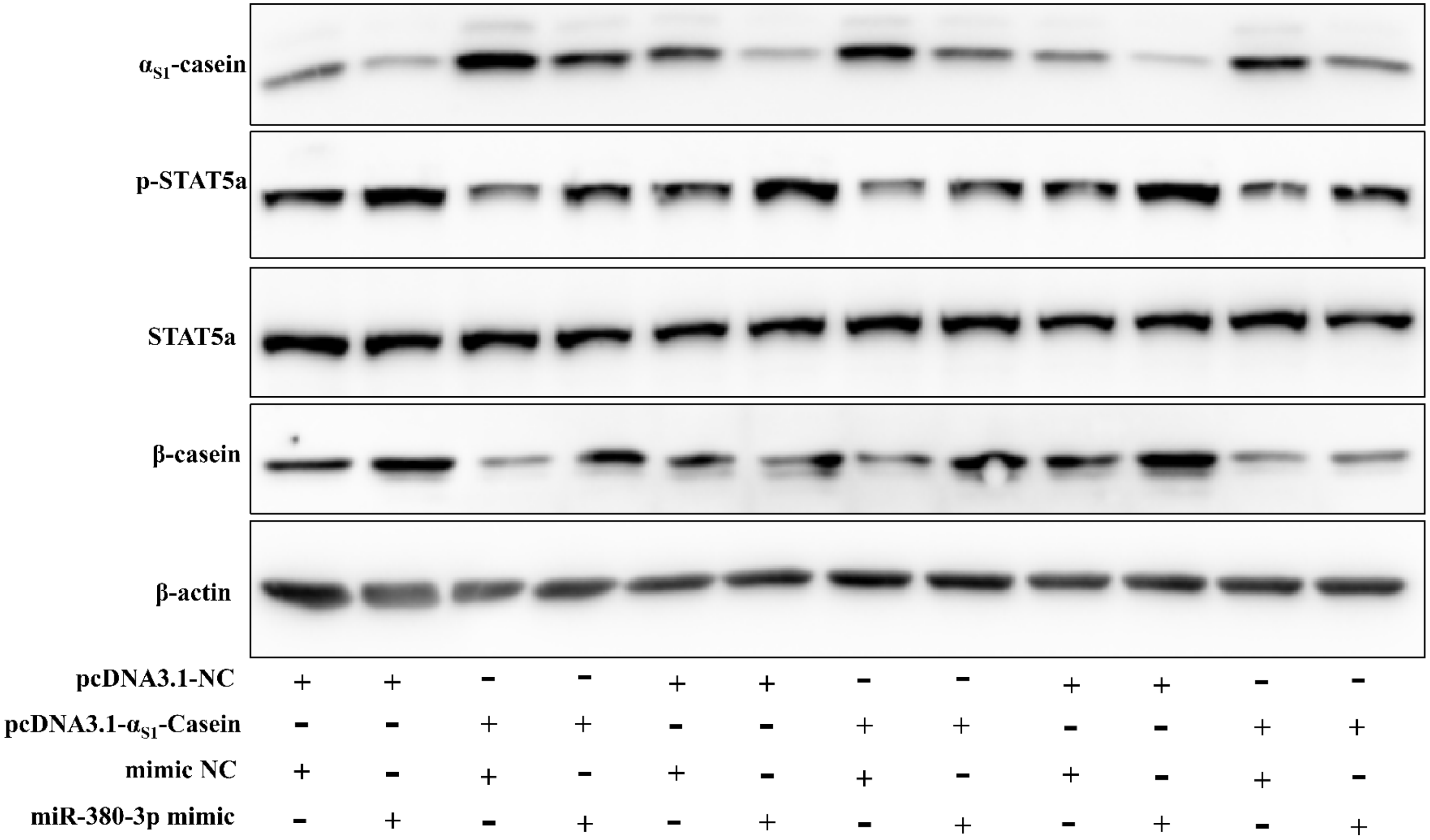

Supplemental Figure S3. The image of the full western blot of Figure 4B. Expression of α_{S1}-casein, p-STAT5a and β-casein in cells co-treated with pcDNA3.1-α_{S1}-casein and miR-380-3p mimic (50 nM) for 48 h.
